# Diabetes Mellitus and Cardiovascular Prevention: The Role and the Limitations of Currently Available Antiplatelet Drugs

**DOI:** 10.1155/2011/250518

**Published:** 2011-06-30

**Authors:** A. Tufano, E. Cimino, M. N. D. Di Minno, P. Ieranò, E. Marrone, A. Strazzullo, G. Di Minno, A. M. Cerbone

**Affiliations:** Regional Reference Centre for Coagulation Disorders, Department of Clinical and Experimental Medicine, University of Naples Federico II, 80131 Naples, Italy

## Abstract

Diabetes mellitus (DM) is associated with macrovascular and microvascular complications. Platelets have a “key role” in atherogenesis and its thrombotic complications in subjects with DM. Moreover, the concomitant presence of multiple “classical” cardiovascular risk factors in diabetic subjects contributes to enhanced atherothrombotic risk. Antiplatelet agents are effective in primary and secondary prevention of arterial thrombosis (cardiovascular events, ischaemic stroke, and peripheral arterial occlusive disease). The role of chronic administration of antiplatelet drugs in primary prevention of arterial vascular events is known to be less clear than in secondary prevention, and, also in diabetic patients, the decision to give primary prophylaxis should be taken on an individual-patient basis, after a careful evaluation of the balance between the expected benefits and the risk of major bleedings. Although, currently, treatment has proven useful in reducing vascular events, diabetic patients continue to have a higher risk of adverse cardiovascular events compared with those in nondiabetic patients. This paper reviews the role of currently available antiplatelet drugs in primary and secondary prevention of vascular events in diabetic patients and the limitations of these drugs, and it discusses the role of novel and more potent antiplatelets and of new agents currently under clinical development.

## 1. Introduction

Diabetes mellitus (DM) is associated with macrovascular and microvascular complications (coronary artery disease, ischemic stroke, peripheral arterial disease, nephropathy, and retinopathy) [1, 2]. 

Platelets have a “key role” in atherogenesis and its thrombotic complications in subjects with DM [3], and the concomitant presence of multiple “classical” cardiovascular risk factors (arterial hypertension, cigarette smoking, and hyperlipidemia) in diabetic subjects contributes to enhanced atherothrombotic risk. 

Platelets from subjects with DM, particularly from those with type 2 diabetes, exhibit increased reactivity. Factors that may contribute to this greater platelet reactivity are not completely elucidated and include metabolic abnormalities as hyperglycemia, hyperlipidemia, insulin resistance, and conditions as oxidative stress, inflammation, and endothelial dysfunction [3].

A close relationship between poor glycemic control and increased platelet activity (estimated by measuring mean platelet volume—MPV—as part of whole blood count) in patients with type 2 DM has been suggested [4]. MPV is considered a marker of platelet function and activation: larger platelets are more reactive and aggregable. They contain denser granules, secrete more serotonin and *β*-thromboglobulin, produce more thromboxane A2 and have more adhesion molecules (like P-selectin and platelet glycoprotein—GP—IIbIIIa), than smaller platelets. It has been shown that MPV is significantly higher in diabetic populations [5]. It is also increased in hypercholesterolemia, metabolic syndrome, acute myocardial infarction, acute ischemic stroke, preeclampsia, and renal artery stenosis [4].

Available antiplatelet agents, such as cyclooxygenase-1 (COX-1) inhibitors (aspirin), ADP P2Y_12_ receptor antagonists, and GP IIb/IIIa receptor inhibitors, are effective and save in the treatment and prevention of thrombotic events, these drugs interfere with the platelet activation process, including adhesion, release, and aggregation.

However, although the currently available treatments have proven to be useful in reducing ischemic events, diabetic patients continue to have a higher risk of adverse events compared with those in non-diabetic patients. The role of novel and more potent antiplatelet strategies, currently under clinical development, seems attractive in diabetic patients.

## 2. Antiplatelet Drugs in Diabetic Patients

Cardiovascular (CV) diseases are the leading cause of morbidity and mortality in diabetic patients. The first evidence of an increased platelet aggregation in diabetics dates back to 1965 [6]. Large clinical trials have shown that antiplatelet agents are effective in the prevention of recurrent cardiovascular events in diabetes. The most prescribed agents are aspirin and clopidogrel, two *cornerstones* of the antiplatelet therapy [7–9].

## 3. Primary Prevention

The Food and Drug Administration has not approved aspirin for use in primary prevention, and the benefit of aspirin among diabetic patients with no previous cardiovascular events is still controversial [10]. In 2007 the American Diabetic Association (ADA) and the American Heart Association (AHA) recommended low doses of aspirin (75–162 mg/day) in primary prevention in diabetics at high cardiovascular risk [11, 12] (i.e., those >40 years of age or with additional risk factors: family history of CV disease, arterial hypertension, cigarette smoking, dyslipidemia, or albuminuria) [13].

In contrast, in the European guidelines aspirin is recommended in primary prevention of ischemic stroke [14].

The results of two recent randomized controlled trials in patients with diabetes raised questions about the efficacy of aspirin in primary prevention [15, 16]. The Japanese Primary Prevention of Atherosclerosis with Aspirin for Diabetes (JPAD) trial was the first prospective trial to evaluate the use of aspirin (81 or 100 mg) in the primary prevention of ischemic events in diabetic type 2 patients (*n* = 2,539), aged 30–85 years, in Japan [15]. In this trial aspirin did not reduce the risk of events in diabetic patients, unless they are aged 65 years and above (*P* = .047 for patients >65 years) [15]. However, aspirin was well tolerated, and there was no significant increase in hemorrhagic complications and hemorrhagic strokes [15].

The Prevention of Progression of Arterial Disease and Diabetes (POPADAD) randomized trial failed to show any benefit of aspirin (100 mg) or antioxidant substances in primary prevention of vascular events in diabetic patients (*n* = 1,276) aged >40 years with an ankle-brachial index ≤0.99, but no symptomatic CV disease. However, this should not be considered a primary prevention trial because the subjects studied had some degree of peripheral arterial disease (PAD) [16].

Moreover, a clear benefit of aspirin (versus placebo) in primary prevention of major cardiovascular events or mortality in diabetes was unconfirmed in a meta-analysis [17].

Finally, the decision to give aspirin must be taken on an individual patient basis, after a careful evaluation of the balance between the expected benefits and the risk of major bleedings [18–20].

Two clinical trials are currently underway, which will provide insights to the usefulness of aspirin in primary prevention in diabetes: A Study of Cardiovascular Events in Diabetes (ASCEND; aspirin 75 mg versus omega-3 fatty acids 1 g), and Aspirin and Simvastatin Combination for Cardiovascular Events Prevention Trial in Diabetes (ACCEPT-D; simvastatin 20–40 mg versus aspirin 100 mg, or simvastatin alone).

## 4. Secondary Prevention

In secondary prevention ADA recommends low-dose aspirin (75–162 mg/d) in diabetic patients affected by vascular events [21]. This position is supported by the results of two large meta-analyses by the Antithrombotic Trialists' Collaboration (ATC), which showed aspirin to be protective in patients at high cardiovascular risk, including those with diabetes [22, 23].

A valid option for patients with aspirin intolerance is represented by ticlopidine and clopidogrel, thienopyridines which affect the adenosine diphosphate (ADP) pathway, by blocking the platelet ADP receptor P2Y_12_ [24].

Clopidogrel has a more favourable safety profile compared with ticlopidine [23]. Bhatt et al. retrospectively analyzed a subgroup of diabetic patients (20% of the study population) in the Clopidogrel versus Aspirin in Patients at Risk of Ischemic Events (CAPRIE) study. Clopidogrel was significantly more effective than aspirin in reducing the risk of ischaemic events in diabetic patients with a history of atherothrombosis [25, 26]. American Diabetes Association guidelines currently recommend the use of clopidogrel in very high-risk diabetic patients, or as an alternative strategy in aspirin-intolerant patients [21].

The Clopidogrel in Unstable Angina to Prevent Recurrent Events (CURE) study (aspirin plus clopidogrel, versus aspirin alone, in patients with unstable angina or non-ST elevation myocardial infarction) confirmed the efficacy of the drug in acute coronary syndromes (ACS), with similar results in diabetic patients. Moreover, the study showed that the event rate was much higher in the diabetic than in non-diabetic group, despite the adjunctive use of clopidogrel [27].

Numerous trials have shown clinical benefits for the Gp IIb/IIIa antagonists (abciximab, eptifibatide, and tirofiban). These drugs significantly reduce the mortality after percutaneous coronary intervention (PCI) in all subgroups of patients considered, including diabetics [28]. These results support the use of Gp IIb/IIIa receptor antagonists in high risk ACS patients, in particular those with diabetes [28].

## 5. Limitations of Currently Available Antiplatelet Drugs and Future Directions

There is a reduced clinical efficacy (“*aspirin resistance*”) of aspirin in diabetic compared with a non-diabetic population. Hyperglycemia may be one of the mechanisms involved in this phenomenon. Increased glycation of platelets and coagulation factors may interfere with acetylation by aspirin [29–35]. This may explain the greater effectiveness of clopidogrel in preventing vascular events in diabetes, as compared with low-dose aspirin [26].

On the other hand, “*clopidogrel resistance*” is a well-described phenomenon in diabetic as well as in non-diabetic patients, with severe clinical consequences (e.g., thromboembolic complications after coronary stent implantation) [36, 37].

Insulin, which interacts with its own receptor on platelet surface, reduces platelet reactivity, by suppressing cAMP and by inhibiting P2Y_12_ signalling. Platelets of diabetic patients are affected by the insulin resistance, which results in an upregulation of the P2Y_12_ pathway and in an increased platelet reactivity [38].

1. Low and high doses of aspirin demonstrate similar reduction in cardiovascular endpoints [22, 23]. Whether the use of higher-doses aspirin in diabetes reduces cardiovascular morbidity is still uncertain [39, 40], but this approach is actually unjustifiable and even unsafe in diabetes, for the increased hemorrhagic and gastrointestinal risk. The available evidence does not consider aspirin doses >100 mg daily as either effective or safe [41].
2. Moreover, aspirin given once daily might be insufficient for patients with increased platelet turnover, as are diabetic patients. “Multiple daily doses,” rather than an increase in a once-daily dose, might be more beneficial in these patients [42–44], as we firstly showed about 30 years ago [45].
3. The association between aspirin and other antiplatelet drugs in some categories of diabetic patients at high thrombotic risk may be considered. In high-risk categories (i.e., ACS) the overall benefits outweigh the increased bleeding risk [46].
4. Several studies have shown the benefit of triple therapy with aspirin, clopidogrel, and cilostazol (a phosphodiesterase III inhibitor), particularly in diabetic patients treated with bare-metal or drug-eluting stents. However, cilostazol therapy has side effects (migraine, palpitations, and gastrointestinal problems) [47].
5. Several studies have focused on how to overcome “clopidogrel resistance” by increasing the dose. In the Optimizing Antiplatelet Therapy in Diabetes Mellitus (OPTIMUS) study, the use of clopidogrel 150 mg/d, chronically, resulted in greater platelet inhibition than clopidogrel 75 mg in type 2 diabetes mellitus [48, 49].
6. New agents are under advanced clinical investigation. These include prasugrel and ticagrelor, which are administered orally, and cangrelor that is administered intravenously, and elinogrel that can be administered in oral and endovenous route. Between orally administered drugs, prasugrel is a third-generation thienopyridine that, like clopidogrel, exerts its antiplatelet effect by P2Y_12_ receptor blockade. However, prasugrel has a more favourable pharmacokinetic profile because it is more efficiently transformed in its active metabolite. In the TRITON-TIMI 38 trial, comparing prasugrel (a 60 mg loading dose and a 10 mg daily maintenance dose) with clopidogrel (a 300 mg loading dose and a 75 mg daily mainteinance dose) in 13,608 patients who underwent percutaneous coronary revascularization, prasugrel significantly reduced the composite primary endpoint of death, nonfatal myocardial infarction and nonfatal stroke (9.9% versus 12.1%, HR 0.81; 95% CI 0.73–0.90; *P* < .001). This clinical benefit was greater in diabetic patients than among patients without diabetes (12.2% versus 17%; HR 0.70, *P* < .001) [50].

Another alternative to aspirin to prevent cardiovascular events in diabetes is considered picotamide (thromboxane A_2_ synthase and thromboxane A_2_ receptors inhibitor). The Drug Evaluation in Atherosclerotic Vascular Disease in Diabetics (DAVID) study is the first trial showing a significant reduction of overall mortality by picotamide versus aspirin in patients at high cardiovascular risk, and in diabetics with peripheral artery disease [51]. These data have to be more extensively evaluated in clinical trial.

## 6. Conclusions

1. Recent findings do not support the use of aspirin in primary prevention of cardiovascular events in diabetics, at difference with secondary prevention. While we await the results of two undergoing primary prevention trials (ASCEND and ACCEPT-D), patients with type 2 DM at high risk of cardiovascular events should be considered for low-doses of aspirin (75–162 mg/day) in primary prevention. A decision should be taken on an individual patient basis.
2. The optimal control of hyperglicemia and other risk factors (arterial hypertension, hyperlipidemia, and cigarette smoking) is necessary to decrease platelet reactivity and to enhance the efficacy of antiplatelet drugs.
3. Recurrent cardiovascular events in diabetic patients, despite antiplatelet therapy, underscore the need of individualized antiplatelet regimens. More specific antiplatelet strategies, as more potent drugs or an association between antiplatelet drugs are warranted in diabetic patients.

## References

[1] Nesto RW (2004). Correlation between cardiovascular disease and diabetes mellitus: current concepts. *American Journal of Medicine*.

[2] Ferroni P, Basili S, Falco A, Davi G (2004). Platelet activation in type 2 diabetes mellitus. *Journal of Thrombosis and Haemostasis*.

[3] Tschoepe D, Roesen P, Schwippert B, Gries FA (1993). Platelets in diabetes: the role in the hemostatic regulation in atherosclerosis. *Seminars in Thrombosis and Hemostasis*.

[4] Demirtunc R, Duman D, Basar M, Bilgi M, Teomete M, Garip T (2009). The relationship between glycemic control and platelet activity in type 2 diabetes mellitus. *Journal of Diabetes and Its Complications*.

[5] Winocour PD (1994). Platelets, vascular disease, and diabetes mellitus. *Canadian Journal of Physiology and Pharmacology*.

[6] Bridges JM, Dalby AM, Millar JHD, Weaver JA (1965). An effect of d-glucose on platelet stickiness. *The Lancet*.

[7] Colwell JA, Nesto RW (2003). The platelets in diabetes: focus on prevention of ischemic events. *Diabetes Care*.

[8] Watala C, Boncler M, Gresner P (2005). Blood platelet abnormalities and pharmacological modulation of platelet reactivity in patients with diabetes mellitus. *Pharmacological Reports*.

[9] Angiolillo DJ (2009). Antiplatelet therapy in diabetes: efficacy and limitations of current treatment strategies and future directions. *Diabetes Care*.

[10] Antithrombotic Trialists’ (ATT) Collaboration (2009). Aspirin in the primary and secondary prevention of vascular disease: collaborative meta-analysis of individual participant data from randomised trials. *The Lancet*.

[11] Buse JB, Ginsberg HN, Bakris GL (2007). Primary prevention of cardiovascular diseases in people with diabetes mellitus: a scientific statement from the American Heart Association and the American Diabetes Association. *Circulation*.

[12] Buse JB, Ginsberg HN, Bakris GL (2007). Primary prevention of cardiovascular diseases in people with diabetes mellitus: a scientific statement from the American Heart Association and the American Diabetes Association. *Diabetes Care*.

[13] Pearson TA, Blair SN, Daniels SR, Eckel RH, Fair JM, Fortmann SP (2002). AHA guidelines for primary prevention of cardiovascular disease and stroke: 2002 update. Consensus panel guide to comprehensive risk reduction for adult patients without coronary or other atherosclerotic vascular diseases (AHA scientific statement). *Circulation*.

[14] Ryden L, Standl E, Bartnik M (2007). Guidelines on diabetes, pre-diabetes, and cardiovascular diseases: executive summary. The task force on diabetes and cardiovascular diseases of the European Society of Cardiology (ESC) and of the European Association for the Study of Diabetes (EASD). *European Heart Journal*.

[15] Ogawa H, Nakayama M, Morimoto T (2008). Low-dose Aspirin for primary prevention of atherosclerotic events in patients with type 2 diabetes: a randomized controlled trial. *Journal of the American Medical Association*.

[16] Belch J, MacCuish A, Campbell I (2008). The prevention of progression of arterial disease and diabetes (POPADAD) trial: factorial randomised placebo controlled trial of Aspirin and antioxidants in patients with diabetes and asymptomatic peripheral arterial disease. *British Medical Journal*.

[17] De Berardis G, Sacco M, Strippoli GFM (2009). Aspirin for primary prevention of cardiovascular events in people with diabetes: meta-analysis of randomised controlled trials. *British Medical Journal*.

[18] Nicolucci A, De Berardis G, Sacco M, Tognoni G (2007). AHA/ADA vs. ESC/EASD recommendations on Aspirin as a primary prevention strategy in people with diabetes: how the same data generate divergent conclusions. *European Heart Journal*.

[19] Colwell JA (2009). Diabetes: does Aspirin use reduce cardiovascular risk in diabetes?. *Nature Reviews Endocrinology*.

[20] Pignone M, Alberts MJ, Colwell JA (2010). Aspirin for primary prevention of cardiovascular events in people with diabetes: a position statement of the American Diabetes Association, a scientific statement of the American Heart Association, and an expert consensus document of the American College of Cardiology Foundation. *Circulation*.

[21] American Diabetes Association (2004). Aspirin therapy in diabetes (position statement). *Diabetes Care*.

[22] Altman R, Carreras L, Diaz R (1994). Collaborative overview of randomised trials of antiplatelet therapy—I. Prevention of death, myocardial infarction, and stroke by prolonged antiplatelet therapy in various categories of patients. *British Medical Journal*.

[23] Antithrombotic Trialist’s Collaboration (2002). Collaborative meta-analysis of randomized trials of antiplatelet therapy for prevention of death, myocardial infarction, and stroke in high risk patients. *British Medical Journal*.

[24] Watala C (2005). Blood platelet reactivity and its pharmacological modulation in (people with) diabetes mellitus. *Current Pharmaceutical Design*.

[25] Gent M (1996). A randomised, blinded, trial of clopidogrel versus Aspirin in patients at risk of ischaemic events (CAPRIE). *The Lancet*.

[26] Bhatt DL, Marso SP, Hirsch AT, Ringleb PA, Hacke W, Topol EJ (2002). Amplified benefit of Clopidogrel versus Aspirin in patients with diabetes mellitus. *American Journal of Cardiology*.

[27] Yusuf S, Zhao F, Mehta SR, Chrolavicius S, Tognoni G, Fox KK (2001). Effects of clopidogrel in addition to Aspirin in patients with acute coronary syndromes without ST-segment elevation. *New England Journal of Medicine*.

[28] Anderson JL, Adams CD, Antman EM (2007). ACC/AHA 2007 guidelines for the management of patients with unstable angina/non-ST-elevation Myocardial Infarction: a report of the American College of Cardiology/American Heart Association Task Force on Practice Guidelines (writing Committee to Revise the 2002 Guidelines for the management of Patients with Unstable angina/ non-ST-elevation Myocardial Infarction) developed in collaboration with the American College of Emergency Physicians, the Society for Cardiovascular Angiographyand Interventions, and the Society of Thoracis Surgeons endorsed by the Amrican Association of cardiovascular and pulmonary Rehabilitation and the Society for Academic Emergency medicine. *Journal of the American College of Cardiology*.

[29] Gasparyan AY, Watson T, Lip GYH (2008). The role of Aspirin in cardiovascular prevention: implications of Aspirin resistance. *Journal of the American College of Cardiology*.

[30] Sanderson S, Emery J, Baglin T, Kinmonth AL (2005). Narrative review: Aspirin resistance and its clinical implications. *Annals of Internal Medicine*.

[31] Angiolillo DJ (2007). Antiplatelet therapy in type 2 diabetes mellitus. *Current Opinion in Endocrinology, Diabetes and Obesity*.

[32] Watala C, Pluta J, Golanski J (2005). Increased protein glycation in diabetes mellitus is associated with decreased Aspirin-mediated protein acetylation and reduced sensitivity of blood platelets to Aspirin. *Journal of Molecular Medicine*.

[33] Di Minno G, Violi F (2004). Aspirin resistance and diabetic angiopathy: back to the future. *Thrombosis Research*.

[34] Ajjan R, Storey RF, Grant PJ (2008). Aspirin resistance and diabetes mellitus. *Diabetologia*.

[35] Cerbone AM, MacArone-Palmieri N, Saldalamacchia G, Coppola A, Di Minno G, Rivellese AA (2009). Diabetes, vascular complications and antiplatelet therapy: open problems. *Acta Diabetologica*.

[36] Geisler T, Anders N, Paterok M (2007). Platelet response to clopidogrel is attenuated in diabetic patients undergoing coronary stent implantation. *Diabetes Care*.

[37] Angiolillo DJ, Fernandez-Ortiz A, Bernardo E (2005). Platelet function profiles in patients with type 2 diabetes and coronary artery disease on combined Aspirin and clopidogrel treatment. *Diabetes*.

[38] Ferreira IA, Mocking AI, Feijge MA (2006). Platelet inhibition by insulin is absent in type 2 diabetes mellitus. *Arteriosclerosis, Thrombosis, and Vascular Biology*.

[39] Colwell JA (2005). Is Aspirin effective in diabetic patients? Yes. *Journal of Thrombosis and Haemostasis*.

[40] Cimminiello C (2005). Is Aspirin effective in diabetic patients? No. *Journal of Thrombosis and Haemostasis*.

[41] Campbell CL, Smyth S, Montalescot G, Steinhubl SR (2007). Aspirin dose for the prevention of cardiovascular disease: a systematic review. *Journal of the American Medical Association*.

[42] Michelson AD, Cattaneo M, Eikelboom JW (2005). Aspirin resistance: position paper of the working group on Aspirin resistance. *Journal of Thrombosis and Haemostasis*.

[43] Davì G, Santilli F (2010). Aspirin as antiplatelet agent in diabetes. PROS-. *European Journal of Internal Medicine*.

[44] Patrono C, Rocca B (2010). The future of antiplatelet therapy in cardiovascular disease. *Annual Review of Medicine*.

[45] Di Minno G, Silver MJ, Cerbone AM, Murphy S (1986). Trial of repeated low-dose Aspirin in diabetic angiopathy. *Blood*.

[46] Wong S, Morel-Kopp MC, Chen Q, Appleberg M, Ward CM, Lewis DR (2006). Overcoming Aspirin resistance: increased platelet inhibition with combination Aspirin and clopidogrel and high dose Aspirin therapy in Aspirin resistant patients with peripheral vascular disease. *Thrombosis and Haemostasis*.

[47] Angiolillo DJ, Capranzano P, Goto S (2008). A randomized study assessing the impact of cilostazol on platelet function profiles in patients with diabetes mellitus and coronary artery disease on dual antiplatelet therapy: results of the OPTIMUS-2 study. *European Heart Journal*.

[48] King SB, Smith SC, Hirshfeld JW, Jacobs AK, Morrison DA, Williams DO (2008). 2007 focused update of the ACC/AHA/SCAI 2005 guideline update for percutaneous coronary intervention: a report of the American College of Cardiology/American Heart Association task force on practice guidelines. *Journal of the American College of Cardiology*.

[49] Angiolillo DJ, Shoemaker SB, Desai B (2007). A randomized comparison of a high clopidogrel maintenance dose in patients with diabetes mellitus and coronary artery disease: results of the optimizing antiplatelet therapy in diabetes mellitus (OPTIMUS) study. *Circulation*.

[50] Wiviott SD, Braunwald E, Angiolillo DJ (2008). Greater clinical benefit of more intensive oral antiplatelet therapy with prasugrel in patients with diabetes mellitus in the trial to assess improvement in therapeutic outcomes by optimizing platelet inhibition with prasugrel-thrombolysis in myocardial infarction 38. *Circulation*.

[51] Neri Serneri GG, Coccheri S, Marubini E, Violi F (2004). Picotamide, a combined inhibitor of thromboxane A2 synthase and receptor, reduces 2-year mortality in diabetics with peripheral arterial disease: the DAVID study. *European Heart Journal*.

